# The Content of Structural and Trace Elements in the Knee Joint Tissues

**DOI:** 10.3390/ijerph14121441

**Published:** 2017-11-23

**Authors:** Wojciech Roczniak, Barbara Brodziak-Dopierała, Elżbieta Cipora, Krzysztof Mitko, Agata Jakóbik-Kolon, Magdalena Konieczny, Magdalena Babuśka-Roczniak

**Affiliations:** 1 Medical Institute, The Jan Grodek Higher Vocational State School, 21 Mickiewicza Str., 38-500 Sanok, Poland; elacipora@interia.pl (E.C.); boras86@wp.pl (M.K.); magda.babuska@vp.pl (M.B.-R.); 2 Department of Toxicology and Bioanalysis, School of Pharmacy with the Division of Laboratory Medicine, Medical University of Silesia, 4 Jagiellonska Str., 41-200 Sosnowiec, Poland; bbrodziak@sum.edu.pl; 3 The Laboratory of Water and Sewage Analyses, Central Mining Institute, Place Gwarków 1, 40-166 Katowice, Poland; kmitko@gig.katowice.pl; 4 Department of Inorganic, Analytical Chemistry and Electrochemistry, Faculty of Chemistry, Silesian University of Technology, 6 B. Krzywoustego Str., 44-100 Gliwice, Poland; agata.jakobik-kolon@polsl.pl

**Keywords:** knee joint tissues, structural and trace elements

## Abstract

Many elements are responsible for the balance in bone tissue, including those which constitute a substantial proportion of bone mass, i.e., calcium, phosphorus and magnesium, as well as minor elements such as strontium. In addition, toxic elements acquired via occupational and environmental exposure, e.g., Pb, are included in the basic bone tissue composition. The study objective was to determine the content of strontium, lead, calcium, phosphorus, sodium and magnesium in chosen components of the knee joint, i.e., tibia, femur and meniscus. The levels of Sr, Pb, Ca, P, Na and Mg were the highest in the tibia in both men and women, whereas the lowest in the meniscus. It should be noted that the levels of these elements were by far higher in the tibia and femur as compared to the meniscus. In the components of the knee joint, the level of strontium showed the greatest variation. Significant statistical differences were found between men and women only in the content of lead.

## 1. Introduction

Environmental exposure, food and drink cause accumulation of some elements in the osseous tissue, which often leads to various illnesses of this tissue. Therefore, the osseous tissue, for which regeneration time is long, can constitute a good reflection of the total level of elements in a human organism [1,2,3].

Calcium can be taken from osseous stock, making the organism nondependent from its supply in a diet. Calcium ions take part in controlling many basic functions of cells and tissues. They are indispensable in activation of many enzymic systems, division of cells, their secretory activities, in muscle cramps, transferring impulses and in many different processes [4,5].

Calcium is essential for bone growth, as it is required for the mineralisation (impregnation of the bone matrix with minerals). An adequate intake of calcium is one of a number of factors that are important for acquiring bone mass and attaining peak bone mass (PBM). Diets containing insufficient amounts of calcium may lead to a low bone mineral density, which may have implications for bone health, notably risk of osteoporosis, in later life [6].

Calcium is a unique cation in living systems because of its dominant role in intra-cellular signaling. Therefore, bone cells that must handle massive amounts of this mineral take special care in its regulation. Sustained elevation of intracellular calcium leads to cell death that is not effectively opposed by the usual regulators of apoptosis [7].

Around 50% of the total content of magnesium is stored in our skeleton, around 40% in muscles and soft tissues [8]. The reasons for the deficit of magnesium in aged people are the decrease of intestinal magnesium absorption and the decrease of the pool of magnesium in bones and excessive loss of it with urine [9,10,11]. Magnesium is a factor stimulating osteoblast mitosis and its lack causes a decrease in the number of these cells. Studies on mice showed that impairment of bone formation due to osteoblasts is an important factor in creating magnesium dependent osteoporosis [12,13]. The shortage of magnesium results in osteoporosis and increase of skeleton fragility [14,15,16].

Sodium, in the form of sodium chloride, increases excretion of calcium with urine (calciuria), which influences the level of calcium in blood and triggers a compensatory reaction that can lead to an enlarged reconstruction of bone and loss of the osseous tissue. With recommended consumption of calcium, it seems that NaCl does not cause harmful effects in bones and calcium metabolism due to the fact that, in order to balance the loss, the absorption of calcium in urine rises. This mechanism can be insufficient when the consumption of calcium is low [17,18,19].

Phosphorus is the major component of all tissues and plays a key role in the mineralization of the skeleton. Its content in bones accounts for 600–700 g [20].

Strontium, due to its marked resemblance to calcium, is mainly accumulated in bones, at an average level of 138 mg/kg. Bones of children are more vulnerable to the accumulation of ^90^Sr than those of adults [21]. The amount of strontium in the skeleton is approximately 0.035 of the calcium content [22]. Strontium is absorbed less effectively from food than calcium and its greater proportion is excreted [23]. It is likely that strontium regulates cellular mechanisms involved in bone cell differentiation. The available in vivo studies indicate that strontium increases bone formation and decreases bone resorption, which leads to bone mass gain and improves mechanical properties of bones [24].

Strontium is a trace element that plays a special role in bone remodeling in the human body, which is associated both with the stimulation of bone formation and reduction in bone resorption [25,26,27]. This mechanism involves an increase in the expression of genes that affect alkaline phosphatase and the activity of mesenchymal stem cells (MSC), as well as inhibition of osteoclast differentiation [28].

The levels of strontium in bones differ according to the anatomical site and bone structure. Greater amounts of strontium occur in the spongy bone than in the cortical bone. The incorporation of strontium into bones, mainly through the exchange on the crystal surface depends on treatment duration, dose, sex and the site in the skeleton [29]. In vitro studies have shown that strontium increases the replication of proteoblastic cells and stimulates the formation of bones in culture cells. Treatment with low doses of strontium administered in the form of strontium chloride or strontium ranelate for 9–26 weeks stimulates bone formation and inhibits bone resorption in humans and rodents [29].

Strontium and calcium not only have chemical and physical properties in common but also show affinity in biological processes. Therefore, the Sr/Ca ratio in bones is frequently mentioned in literature and has been used by a few authors [23,30,31] to define strontium absorption, accumulation or deposition in bones in relation to calcium.

The study objective was to determine the content of strontium, lead, calcium, phosphorus, sodium and magnesium in chosen components of the knee joint, i.e., tibia, femur and meniscus, using the correlation analysis. The investigations were performed to determine in which tissue the concentrations of the elements studied are the highest and the lowest. The Sr/Ca, Ca/P, Pb/Ca ratios, which are frequently discussed in literature [32,33,34,35,36,37,38], were calculated to determine the element content.

## 2. Material and Methods

The study material included parts of the knee joint obtained during endoprosthesoplasty in the Dr. Janusz Daab Hospital of Trauma Surgery in Piekary Śląskie. Biological samples were obtained from patients living in Silesia Province. Samples were collected from 50 patients, 36 women and 14 men. In 26 patients, the right leg, and, in 24 patients, the left leg were involved. The mean age of the whole study population was 67.5 years, being slightly lower in women—67.2 years—than in men—68.1 years. In the study group, patients complained of pain of 10 years’ duration. A detailed description of the test group patients is shown in Table 1.

The study was approved by the Bioethics Committee No. 2/2013 of 18 June 2013. Degenerative disease of the knee joint and considerable pain were indications for this type of procedure. Surgeries were performed in subarachnoid anesthesia, with patients in the prone position. Esmarch bandage was used for exsanguination of the limb. The frontal surface of the knee joint was exposed following standard preparation of the operation field (applying antiseptic and aseptic techniques) with straight midline incision. The joint was opened at the medial side and the hypetrophic synovium was removed. Using ZIMMER instrumentation (Zimmer Biomed, Warsaw, IN, USA), the femoral part of the knee joint was prepared, by preparing the distal femur and performing femoral epicondyle osteotomy. Next, damaged menisci were removed, and, using ZIMMER instrumentarium the tibial part was prepared (resection of the tibial plateau). In this way, the osseous components, cartilages and parts of menisci were used for measurements.

The material samples were described and stored in modified polyethylene containers, in a freezer at a temperature of −22 °C.

Tissue samples with a known mass were mineralized using 4 cm^3^ of spectrally pure HNO_3_ (V) (Supra pure, Merck, Dormstadt, Germany) in a microwave mineralizer Magnum II (Ertec, Wrocław, Poland). The samples were placed one by one in a Teflon vessel and mineralization was added. Mineralization was a two-stage procedure. The first stage lasted 2 min at 20 bar max pressure and 255 °C max temperature, whereas the second stage was of 6 min duration at 45 bar max pressure and 285 °C max temperature. The post-mineralization solution was transferred to a 25 cm^3^ flask and then diluted to the ml mark with redistilled water.

The content of calcium, magnesium, phosphorus, sodium and lead in mineralized samples was determined using inductively coupled plasma atomic emission spectrometry (ICP-AES). A Varian 710-ES spectrometer equipped with a OneNeb nebulizer was utilized. The following parameters were used: RF power 1.0 kW, plasma flow 15 L/min, auxiliary flow 1.5 L/min, nebulizer pressure 210 kPa, pump rate 15 rpm, emission lines of Ca: λ = 211.276 nm, Mg: λ = 280.270 and 285.213 nm, P: λ = 213.618 and 214.914 nm, Na: λ = 566.348 and 589.592 nm, Pb: λ = 220.353 nm. The calibration curve method was applied. The standard solutions of 1 mg/mL (Millipore SAS, Molsheim, France) as well as deionized water (Elix Essential 10, Merck Millipore, Burlington, MA, USA) were used. The results are an average of the concentrations obtained for all analytical lines used for the element, with standard deviation not exceeding 1.5%. The accuracy of the analysis was controlled using Standard Reference Material 1400 Bone Ash (NIST-National Institute of Standards and Technology).

The content of strontium in samples was determined by means of plasma emission spectrometry technique (ICP-OES), using a PerkinElmer Optima 5300DV spectrometer (horizontal plasma, Echelle-type diffraction net, a simultaneous semiconductor detector SCD). Measurements were performed in the plasma axis, usually using two spectral lines for the element studied and two-point background correction. Robust plasma conditions were applied, i.e., plasma power of 1500 W and gas flow of 0.6 L/min. The automatic time of integration was set at 2–10 s. and the measurement was repeated twice in one sample administration. The sample was administered to the plasma using a shunt pump, through a MiraMist nebuliser in connection with a cyclonic spray chamber and a ceramic injector. The sample flow was 2 mL/min.

The statistical analysis was made using the Statistica Pl. 12 software (StatSoft, Crocow, Poland). Non-parametric tests were used to assess the importance of differences between groups of results (Mann–Whitney U test for two samples, ANOVA rang Kruskal–Wallis test for multiple samples), significance level of *p* < 0.05 was assumed to be statistically significant. Moreover, Spearman’s rank correlation was determined, significant correlation coefficients occurred at a probability level of *p* < 0.05.

## 3. Results

The mean Sr content in the knee joint was 17.50 mg/kg, Pb 1.88 mg/kg, Mg 1024.56 mg/kg, Ca 80.04%, P 36.04%, Na 4.29%. The highest variability was observed in the content of lead (110%), the lowest in sodium—45%.

No statistically significant differences were noted between women and men in the levels of Sr, Ca, P, Na and Mg, but they were observed for lead (Mann–Whitney U test, *p* = 0.05). The content of these elements was slightly higher in men than in women, except for magnesium, the level of which was nearly identical in both sexes. 

Statistically significant differences were found in the content of Sr, Pb, Ca, P, Na, Mg in the respective components of the hip joint (ANOVA Kruskal–Wallis test *p* < 0.001). The content of strontium in the tibia and femur was 26.64 and 24.60 mg/kg, respectively, and in the meniscus 1.44 mg/kg. Lead content in the meniscus was 0.32 mg/g, in the tibia 2.67 mg/g, and in the femur 2.64 mg/g. Calcium content was the highest in the tibia 12.26% and femur 11.25%, being approximately 23 times lower in the meniscus (5.08 mg/kg). Likewise, the level of phosphorus was the highest in the tibia (5.53%) and femur (5.06%), the lowest in the meniscus (2.21 mg/kg). The levels of sodium in the tibia, femur and meniscus were 0.55%, 0.52% and 0.22%, respectively. The levels of magnesium were found to be 1554.89 mg/kg in the tibia, 1419.34 mg/kg in the femur and 99.46 mg/kg in the meniscus.

The levels of Sr, Pb, Ca, P, Na and Mg investigated in the study with regard to the part of the hip joint and sex have been presented in Table 2. They were the highest in the tibia in both men and women, whereas they were the lowest in the meniscus. It should be noted that the levels of these elements were by far higher in the tibia and femur as compared to the meniscus. Statistically significant differences between men and women were observed only in the tibia and referred to the content of lead (Mann∓Whitney U test, *p* = 0.011).

Smoking was not found to have any impact on the content of strontium, lead, phosphorus, sodium and magnesium. Of them, only the level of strontium was higher in smokers, whereas the levels of lead, calcium, phosphorus, sodium and magnesium were higher in non-smokers (Table 3). No differences were also noted between the respective components of the knee joint and sex or smoking.

The levels of the elements studied in the bone tissue were much higher than in the connective tissue (P—24×, Ca—23×, Sr—18×, Mg—15×, Pb—8× and Na—3×). This indicates that phosphate and strontium, similarly to calcium, are characterized by high accumulation potential in the bone tissue. The level of phosphate was approximately 24 times higher and the level of strontium 18 times higher in the bone tissue as compared to the connective tissue. This confirms that strontium in its properties resembles calcium and is able to accumulate in the hard bone tissue but not in the soft connective tissue. There was eight times more lead in the bone tissue than in the connective tissue.

**Figure 1:**
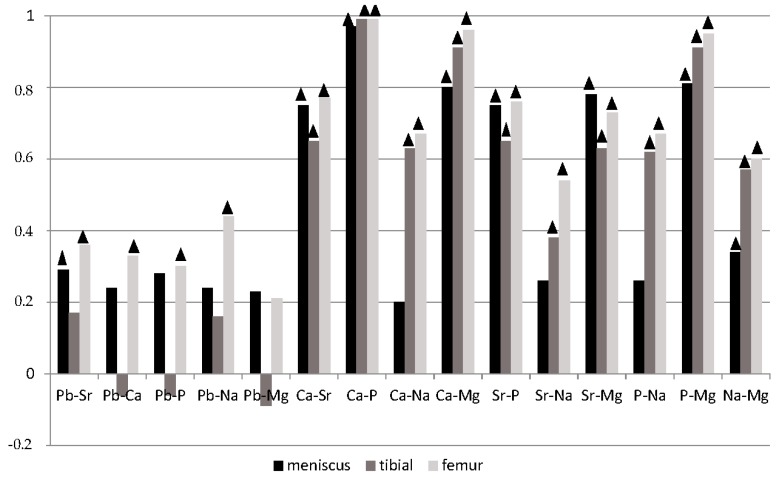
The Spearman’s correlation coefficients for strontium, lead, magnesium, sodium, phosphorus and calcium in tissues of the knee joint (▲ correlation statistically significant *p* < 0.05).

The Spearman correlation analysis (*p* < 0.05) showed many statistically significant synergistic correlations between the elements studied (Figure 1).

Most significant correlations were noted in the femur, where strontium, calcium, phosphate and sodium correlated with all the elements studied. In the tibia, lead showed no correlation at all. The fewest correlations were noted in the meniscus, with more correlations found for strontium and magnesium and the fewest for lead and sodium.

The Ca/P ratios in the same site of the bones differ significantly among various species. In the hind tibiae of the lamb, rabbit and rat, the mean Ca/P values were 1.35, 1.75 and 1.94, respectively. The study revealed a significant difference (*p* < 0.001) in the Ca/P ratio between normal bone and the bone affected by osteoporosis. Moreover, the results presented also confirm the relationship between bone mass loss and a reduced Ca/P ratio [39].

The highest Sr/Ca ratio was found in the meniscus (0.305 × 10^−3^ in the whole population). On the other hand, the Ca/P ratio was the lowest in the meniscus as compared to the other components of the knee joint. In the femur, the Sr/Ca and Ca/P ratios were higher in comparison with the tibia. A comparison between women and men showed a higher Sr/Ca ratio in women but a higher Ca/P ratio in men—Table 4.

## 4. Discussion

The bone composition can be described with reference to the mineral phase, hydroxyapatite and the organic phase. Relative proportions of these components differ according to age, sex, disease and treatment. Pharmacotherapy and its type can alter bone composition [40].

Up to now, research into the content of elements in the knee joint has been uncommon, which is associated with the fact that, in Poland, knee replacement surgery is seldom performed. Krachler et al. [41] and Naza [42] determined the content of elements in blood serum in patients with knee osteoarthritis (OA) in the fluid from the knee joint [43]. Patients with OA had higher levels of Cu, higher Cu/Zn ratio and lower concentrations of Zn and Se in the serum, and these alterations were correlated with the disease duration and intensity [42]. Lanocha-Arendarczyk et al. [44] investigated the content of elements in the human tibia plateau from patients after knee surgery. In her study, the content of strontium in the tibia was 44.10 µg/g as compared to 26.64 µg/g in our research. On the other hand, the content of lead was higher in our study (2.67) than in the research conducted by Lanocha-Arendarczyk et al. [44]. The differences seem to depend on the place of residence of the study participants. Lanocha-Arendarczyk et al. [44] examined patients from the north of Poland, i.e., poorly industrialized region, whereas patients from our study inhabited the highly industrialized region of Upper Silesia. Both in our study and in the research conducted by Lanocha-Arendarczyk et al. [44], women showed higher levels of strontium and lower concentrations of lead as compared to male patients. However, statistically significant sex-dependent differences in lead content were confirmed only in our study (Mann–Whitney U test, *p* = 0.011).

The studied parts of the knee joint were characterized by a wide range of elements, from the smallest amount of lead in the meniscus (0.11 µg/g) to the highest level of calcium in the tibia (19.23%). The highest variability in the tibia and femur was observed for lead, and then for strontium, phosphate and calcium. Sodium was the most stable element. On the other hand, calcium and phosphate showed the greatest variability in the meniscus, with sodium being the most stable.

Zioła-Frankowska et al. [45] investigating the hip joint confirmed the occurrence of correlations of calcium with Mg, P, Sr, Na, and of lead with Mg, P, Na, Ca, just like in the knee joint. Helliwell et al. [46], in a study on the content of elements in the femoral head confirmed the correlation of calcium with sodium (*r* = 0.83), magnesium (*r* = 0.89) and strontium (*r* = 0.68). Kuo et al. [3] observed the co-occurrence of calcium with Pb (*r* = 0.55) and Mg (*r* = 0.72). Milachowski [47] confirmed the correlation between magnesium and calcium in the femoral head.

In the current study, the content of magnesium was similar to the results reported by Nowakowski et al. [48] (949.1 mg/kg in the intervertebral discs vs. 1024.56 mg/kg in our study).

In the bones obtained from inhabitants of Tarragona (Spain), the content of lead was 1.79 µg/g (ww), which was similar to our findings—1.88 µg/g [49]. In the bones of men, the content of lead was higher (2.56 µg/g) than in women (2.16 µg/g), like in our study (2.58 mg/kg and 1.60 mg/kg, respectively).

The level of strontium was by far lower (18.64 mg/kg) as compared to the results reported by Lanocha-Arendarczyk et al. [44] (44.10 µg/g).

The comparison of our findings with those reported from excavated bones [50] showed substantially higher levels of lead (6.85 µg/g), strontium (80.51 µg/g), calcium (246 mg/g), phosphate (119 mg/g), magnesium (2.85 mg/g) and sodium (5.18 mg/g) in the excavated bones. This may be the effect of migration of the elements from soil to bones.

The content of strontium determined in the femoral head by Budis et al. [51] was 48.5 mg/kg in spongy bone and 26.7 mg/kg in cartilage with adjacent compact bone. These values were considerably higher than in the tibia (26.64 mg/g), in the femur (24.60 mg/g) and in the meniscus (1.44 mg/g).

The values of strontium were many times higher in comparison with those reported by Zaichick et al. [52] in the ribs—291 mg/kg—and in the femoral neck—288 mg/kg [53].

The levels of calcium in the femur and tibia were similar to those found in the hip joint in a study conducted in Poland by Zioła-Frankowska et al. [45]. The level of calcium was lower (136.71 g/kg) in the femoral head than in the femoral neck (157.21 g/kg) (in our study—tibia 122.57 g/kg and femur 112.46 g/kg) [45].

The level of phosphate in the hip joint was slightly higher than in our study; it was lower in the femoral head—62.72 g/kg—than in the femoral neck—70.65 g/kg.

The levels of sodium and magnesium in the knee joint were the same as in the hip joint (hip joint Na 1.49 vs. 1.52 g/kg, Mg 5.37 vs. 5.08 g/kg) [36].

The content of strontium in the hip joint according to Zioła-Frankowska et al. [45] was much lower (45.65 µg/g) than in our study, including 25.62 mg/kg in the tibia and 1.44 mg/kg in the meniscus on average. On the contrary, the content of lead in our study was higher than in the study conducted by Zioła-Frankowska et al. [45] (1.88 vs. 1.12 mg/kg).

According to literature data, the content of calcium in hydroxyapatite was 39.9%, phosphate 18.5% and the Ca/P ratio 2.16 [32]. Apart from hydroxyapatite, bones also contain collagen, bone marrow, fats, protein and water. Therefore, the levels of Ca and P in the human bone does not correspond to the theoretical values in hydroxyapatite. Investigations conducted by Zaichick and Tzaphlidou [32] revealed that the Ca content in the bone ranged between 4.6% and 28.4%, and P content from 2.6 to 20.0%. In bones of other species, the levels of Ca and P determined by means of various methods were 18.5–62% and 8.7–27%, respectively [54,55]. Since bones are the material that are structurally adapted to perform various functions, the exact composition may differ depending on sex, age, type of bone and the site in the body.

The levels of the elements studied were similar to those found in the ribs by Takata et al. [55]—calcium (15.08% vs. 11.76%), phosphate (6.69% vs. 5.30%), sodium 4.28 vs. 5.37 g/kg), and magnesium (1.78 vs. 1.49 g/kg). The differences were the greatest for strontium (76.25 µg/g in the Takata et al. [55] study, 25.62 µg/g in the current study).

Similar results were reported by Scancar et al. [56] in the human iliac crests, e.g., strontium 23.65 mg/kg, lead 1.20 mg/kg and calcium 114.51 g/kg.

In the bones from industrialized Taiwan, the levels of magnesium (3.01 µg/g) and lead (7.10 µg/g) were higher than those determined in the current study (Mg—1.02, Pb—1.88 µg/g), whereas calcium content was at the same level (82.00 vs. 80.04) throughout the hip joint [3].

The content of elements in the femoral head in patients from the south of Poland was similar to the level noted for the tibia: Ca 17.01% vs. 12.26%, Pb 2.76 vs. 2.67 µg/g, and Mg 1.76 vs. 1.55 g/kg [57].

According to Milachowski [47], calcium content was 196.9 g/kg in the femoral head in the articular surface, 132 g/kg in the cortical bone and 94.7 g/kg in the spongy bone. Our results concerning calcium were similar. In a study conducted by Milachowski [47], the level of magnesium was substantially higher than in our research, being 2.37 g/kg in the articular surface of the femoral head, 2.55 g/kg in the spongy bone and 3.37 g/kg in the spongy bone.

The Ca/P ratio was presented in a study conducted by Tzaphlidou and Zaichick [33,58], being 2.17 in the cortical bone from the human femoral neck and 2.22 throughout the hip joint. Interestingly, the ratio was lower in the meniscus (2.08) in comparison with the femur (2.23) and tibia (2.22). This results from the fact that the level of calcium in the meniscus was 23-fold lower than in the femur and tibia, whereas the content of phosphate was only eight times lower. The Ca/P ratio in the ribs of healthy individuals was 2.33, being higher in men (2.35) than in women (2.31) [35]. In the bones from excavations in Finland, the Ca/P ratio was 2.09, which is very similar to the level obtained for the meniscus in our study [34].

The analysis of the Ca/P ratio in various types of bones is presented in terms of its variability according to the type of bone, site of sample collection and in patients with bone disorders. The determination of the Ca/P ratio can be a sensitive measurement of mineral alterations within the bone. It has been known that mechanical resistance depends first of all on the condition of the cortical bone [35,59]. Mechanical control of the resected necks of the femur showed that the cortical bone accounted for 40–60% of the total resistance of the femur [39].

The Ca/P and Ca/Sr ratios were calculated by Fabig and Herrmann [31], who investigated human bones from archaeological excavations. The Ca/P ratio ranged from 2.14 to 2.34 (in our study 1.99–2.25) [31]. The ratio of 1.99 was found in the meniscus, whereas it was 2.21–2.23 in the femur and tibia. Marked differences were found in the Sr/Ca ratios between our study (0.2–0.35 × 10^−3^) and the results reported by Fabig and Herrmann [31] (0.44–0.95 × 10^−3^).

## 5. Conclusions

The femur and the tibia contained 24 times more phosphorus, 23 times more calcium, 18 times more strontium, 15 times more magnesium, eight times more lead and three times more sodium as compared to the meniscus.

In the components of the knee joint, the level of strontium showed the greatest variation.

Significant statistical differences were found between men and women only in the content of lead.

No effect of smoking was noted regarding the content of strontium, lead, calcium, phosphorus, sodium and magnesium in the tissues examined.

## Figures and Tables

**Table 1:** Information about the study group patients (AM-arithmetic mean, SD-standard deviation).

Parameters	Whole Population *n* = 50	Females *n* = 36	Males *n* = 14
Age (years)			
AM ± SD	67.46 ± 7.11	67.22 ± 7,09	68.07 ± 7.20
range	54–78	54–78	56–78
Body weight (kg)			
AM ± SD	83.54 ± 14.56	81.45 ± 14.19	88.58 ± 14.56
range	54–115	54–115	66–108
Height (cm)			
AM ± SD	164.37 ± 9.32	160.24 ± 6.14	174.33 ± 8.11
range	149–189	149–173	165–189
Smokers (*n*, %)			
- nonsmokers	20 (40%)	19 (38%)	1 (2%)
- smokers	21 (42%)	10 (20%)	11 (22%)
- smokers in the past	9 (18%)	5 (10%)	4 (8%)
Place of residence (%)			
Village	11 (22%)	7 (14%)	4 (8%)
Town	39 (78%)	29 (58%)	10 (20%)
Knee (%)			
Left	24 (48%)	18 (36%)	6 (12%)
Right	26 (52%)	18 (36%)	8 (16%)
Beginning pain (years, %)			
<5	16 (32%)	11 (22%)	5 (10%)
<10	21 (42%)	15 (30%)	6 (12%)
>10	13 (26%)	10 (20%)	3 (9%)
Earlier knee endoprosthesis (%)			
Yes	13 (26%)	10 (20%)	3 (6%)
No	37 (74%)	26 (52%)	11 (22%)
Degenerative changes in the other knee (%)			
Yes	33 (66%)	23 (46%)	10 (20%)
No	17 (34%)	13 (26%)	4 (8%)
Contact with chemicals in the workplace			
(factory PVC-polyvinylchloride, zinc smelter) (%)	3 (6%)	1 (2%)	2 (4%)

**Table 2:** Statistical characteristics for concentration of strontium, lead, magnesium, sodium, phosphorus and calcium in tissues of the knee joint (AM—arithmetic mean; SD—standard deviation; Med—median; CV—coefficient variability; M–W—Mann–Whitney U test; NS—non-significant).

	Meniscus				Tibia				Femur			
	AM ± SD	Med.	Range	CV	AM ± SD	Med.	Range	CV	AM ± SD	Med.	Range	CV
Female												
Sr (mg/kg)	1.26 ± 0.77	1.06	0.27–3.46	61	26.69 ± 10.44	25.52	11.47–56.73	39	24.57 ± 10.13	23.26	8.82–55.98	41
Pb (mg/kg)	0.24 ± 0.18	0.17	0.11–1.05	77	2.16 ± 1.66	1.73	0.42–7.02	77	2.41 ± 1.97	1.74	0.12–9.40	82
Mg (mg/kg)	78.74 ± 36.88	68.27	37.78–209.94	47	1549.45 ± 366.84	1562.44	889.72–2192.12	24	1453.84 ± 414.94	1437.11	756.10–2194.35	29
Na (%)	0.18 ± 0.07	0.18	0.10–0.46	37	0.54 ± 0.09	0.54	0.34–0.72	17	0.53 ± 0.08	0.52	0.32–0.68	16
P (%)	0.19 ± 0.20	0.14	0.03–0.96	103	5.51 ± 1.50	5.53	2.88–8.62	27	5.00 ± 1.60	4.7	2.53–7.65	32
Ca (%)	0.34 ± 0.26	0.27	0.04–1.06	76	12.21 ± 3.29	12.19	6.33–19.23	27	11.12 ± 3.41	10.42	5.89–17.15	31
Male												
Sr (mg/kg)	1.93 ± 1.80	1.25	0.56-5.69	94	26.52 ± 9.76	27.33	12.57–47.09	37	24.66 ± 11.08	27	8.55–40.28	45
Pb (mg/kg)	0.53 ± 0.63	0.19	0.13–1.73	120	3.99 ± 2.61	3.29	0.50–9.75	65	3.22 ± 2.59	2.9	0.13–10.03	80
Mg (mg/kg)	152.74 ± 193.64	65.52	41.02–612.32	127	1568.89 ± 397.14	1591.00	955.84–2195.89	25	1330.60 ± 547.40	1471.24	370.02–2138.12	41
Na (%)	0.28 ± 0.21	0.17	0.12–0.70	74	0.58 ± 0.14	0.57	0.41–0.81	23	0.52 ± 0.16	0.54	0.19–0.85	32
P (%)	0.30 ± 0.42	0.12	0.03–1.18	139	5.59 ± 1.87	5.96	3.00–8.80	33	5.21 ± 1.92	5.51	2.10–7.83	37
Ca (%)	0.25 ± 0.23	0.2	0.01–0.92	93	12.37 ± 3.91	13.17	6.92–18.93	32	11.56 ± 4.19	12.25	5.17–17.40	36
Total												
Sr (mg/kg)	1.44 ± 1.17	1.07	0.27–5.69	81	26.64 ± 10.15	25.9	11.47–56.73	38	24.60 ± 10.29	23.95	8.55–55.98	42
Pb (mg/kg)	0.32 ± 0.38	0.18	0.11–1.73	121	2.67 ± 2.11	2.1	0.42–9.75	79	2.64 ± 2.16	2.05	0.12–10.03	82
Mg (mg/kg)	99.46 ± 109.75	67.95	37.78–612.32	110	1554.89 ± 371.55	1572.40	889.72–2195.89	24	1419.34 ± 453.43	1443.62	370.02–2194.35	32
Na (%)	0.21 ± 0.13	0.18	0.10–0.70	61	0.55 ± 0.11	0.54	0.34–0.81	19	0.52 ± 0.11	0.54	0.19–0.85	21
P (%)	0.22 ± 0.28	0.13	0.03–1.18	125	5.53 ± 1.60	5.58	2.88–8.80	29	5.06 ± 1.68	4.84	2.10–7.83	33
Ca (%)	0.31 ± 0.25	0.26	0.01–1.06	80	12.26 ± 3.43	12.45	6.33–19.23	28	11.25 ± 3.61	10.7	5.17–17.40	32

**Table 3:** Statistical characteristics for concentration of strontium, lead, magnesium, sodium, phosphorus and calcium in tissues of the knee joint people non-smoker and smoker (AM—arithmetic mean; SD—standard deviation; Med—median; CV—coefficient variability).

	AM ± SD	Med.	Range	CV
non-smoker				
Sr (mg/kg)	17.20 ± 14.25	17.77	0.27–56.73	83
Pb (mg/kg)	1.99 ± 2.34	1.35	0.11–10.03	118
Mg (mg/kg)	1019.25 ± 732.19	1158.10	37.78–2192.12	72
Na (%)	0.42 ± 0.19	0.49	0.10–0.73	45
P (%)	3.58 ± 2.74	3.70	0.03–8.80	77
Ca (%)	7.90 ± 6.14	8.55	0.04–19.23	78
smoker				
Sr (mg/kg)	18.88 ± 14.14	20.90	0.61–47.09	75
Pb (mg/kg)	1.72 ± 1.61	1.13	0.12–5.46	94
Mg (mg/kg)	976.41 ± 697.07	1165.12	47.72–2195.89	71
Na (%)	0.40 ± 0.20	0.44	0.12–0.81	51
P (%)	3.33 ± 2.49	3.98	0.07–7.67	75
Ca (%)	7.35 ± 5.46	8.77	0.17–16.42	74

**Table 4:** The ratios of the elements in tissues of the knee joint.

		Sr/Ca (×10^−3^)	Ca/P	Pb/Ca
Men	Femur	0.22	2.22	0.03
Men	Meniscus	0.28	2.14	0.10
Men	Tibial	0.21	2.21	0.03
Women	Femur	0.24	2.23	0.02
Women	Meniscus	0.34	1.99	0.07
Women	Tibial	0.23	2.22	0.02
Women		0.24	2.21	0.02
Men		0.22	2.21	0.03
Total		0.23	2.21	0.02
Femur		0.23	2.23	0.02
Meniscus		0.31	2.08	0.10
Tibial		0.23	2.22	0.02
Smoker		0.22	2.21	0.03
Non-smoker		0.26	2.20	0.02
People >60 years of age		0.23	2.22	0.01
People 61–70 years of age		0.20	2.29	0.02
People <71 years of age		0.22	2.25	0.01
